# PAHs: Choi et al. Respond

**DOI:** 10.1289/ehp.0800445R

**Published:** 2009-04

**Authors:** Hyunok Choi, John Spengler, Frederica P. Perera

**Affiliations:** Department of Environmental Health, Harvard School of Public Health, Boston, Massachusetts, E-mail: hchoi@hsph.harvard.edu, E-mail: Spengler@hsph.harvard.edu; Columbia Center for Children’s Environmental Health, Mailman School of Public Health, Columbia University, New York, New York, E-mail: fpp1@columbia.edu

We appreciate the comments of Fowler et al. In their letter, they compared the maximal maternal inhalation level of polycyclic aromatic hydro carbons (PAHs) in Krakow, Poland (Choi et al. 2008), with the mean fetal liver dose in Aberdeen, United Kingdom (Fowler et al. 2008). The goal of this comparison seems to have been to examine whether the fetal exposure concentration following transplacental transfer of the PAHs is higher than the airborne concentration for the mother. This is an important question because in epidemiologic investigations of developmental and health consequences of prenatal PAH exposure, researchers often assume comparability in maternal exposure level and fetal exposure dose. Fowler et al. propose two inferences in their comments: *a*) the fetal liver exposure concentration resulting from transplacental transfer of the PAHs is higher than the maternal exposure concentration of airborne PAHs; and *b*) the relative proportion of individual PAHs for the fetal liver might be different from those experienced by the mothers. However, the two studies differ in several important aspects. Direct fetal–maternal comparison based on combining the data from the two studies as proposed by Fowler et al. is problematic for the following reasons.

First, in our study (Choi et al. 2008) we targeted nonsmoking pregnant women with no known risks of adverse birth outcomes in Krakow, Poland. To preclude the possibility of confounding by heavy environmental tobacco smoke (ETS) exposure or unreported maternal cigarette smoking, we further restricted the mother–newborn pairs to those with umbilical cord cotinine concentrations < 25 ng/mL in our analysis of the birth outcomes. Within our Polish cohort, mean (± SD) newborn and maternal plasma cotinine levels were 0.32 ± 0.88 and 0.28 ± 0.24 ng/mL, respectively (Choi et al. 2006). Fetal cotinine levels reported by Fowler et al. (2008) for the smoking mothers (45.2 ± 2.5 ng/mL, mean ± SE) were about 75-fold higher than those for the nonsmoking mothers (0.6 ± 1.9 ng/mL). In contrast, the fetal liver concentrations of benz[*a*]anthracene, benzo[*ghi*] perylene, benzo[*a*]pyrene (BaP), chrysene, dibenzo[*a,h*]anthracene, and indeno[1,2,--*cd*]pyrene for the smoking mothers are lower than those of the nonsmoking mothers by to 18–75% [Table 3 of Fowler et al. (2008)]. Lack of correlation between cotinine and PAH levels in the nonsmoking women raise doubt about the appropriateness of the compari son based on their control group.

Second, major sources of the PAHs in Krakow were coal- and diesel-combustion (Choi et al. 2008). In contrast, the relative proportions of the fetal liver PAHs from the smoking and the nonsmoking women reported by Fowler et al.(2008) are almost identical. This suggests that major sources of PAHs in this study are maternal smoking or intensive ETS exposure. In their letter, Fowler et al. observed that the relative proportion of the maternal airborne exposure (from our study) is very different from the similar proportions in the fetal liver. They interpret this as very different proportions of PAHs accumulating in the liver following the maternal inhalation. However, we suspect that the difference in relative proportions of maternal air and fetal liver PAHs is likely due to the differences in the sources of the PAHs between the two studies, as well as to differential accumulation.

Third, it is unknown whether the demographic and socioeconomic characteristics of the maternal cohorts of the two studies are similar enough to support the comparison of the maternal airborne concentration with the fetal liver level. The maternal cohort of Fowler et al. (2008) elected medical abortion; about half of these women smoked intensively during their pregnancy. In contrast, the women in our cohort (Choi et al. 2008) successfully carried their pregnancies and were more likely to engage in healthy behaviors. Therefore, residual confounding by the maternal behaviors (such as micro nutrient intake during pregnancy), socioeconomic status, and genetic polymorphisms might have further contributed to the differences in maternal air and fetal liver dose.

A substantial body of evidence suggests that fetuses are much more susceptible to PAH-induced carcinogenesis than are adults (Rice 1982; Soyka 1980), despite the likelihood that the transplacental dose of PAHs to the fetus is at least an order of magnitude lower than the maternal tissue levels (Neubert and Tapken 1988; Withey et al. 1993). Perera et al. (2004) reported that fetal PAH–DNA adduct levels were generally similar to those in the mothers; among ETS exposed mother–newborn pairs, the mean (± SD) maternal BaP–DNA adduct level was 0.23 ± 0.16 per 10^8^ nucleotides, and the fetal level was 0.24 ± 0.15 per 10^8^ nucleotides. Among the non-ETS exposed pairs, the mean maternal BaP–DNA adduct level was 0.21 ± 0.12 per 10^8^ nucleotides, and the fetal level was 0.24 ± 0.15 per 10^8^ nucleotides (Perera et al. 2004).

The issue raised by Fowler et al. in their letter is important because little is known about the actual concentration or type of PAHs that reach the fetus following maternal inhalation. Determining the actual fetal tissue dose is critical in understanding the sources of fetal vulnerability as well as the health effects of their exposure. However, the lack of comparability in maternal populations of the two studies does not allow valid estimation of the magnitude of maternal–fetal level difference. We believe the concentration of fetal tissue accumulation in humans following maternal inhalation of the PAHs needs further investigation.

## References

[b1-ehp-117-a140a] Choi H, Jedrychowski W, Spengler J, Camann DE, Whyatt RM, Rauh V (2006). International studies of prenatal exposure to polycyclic aromatic hydrocarbons and fetal growth. Environ Health Perspect.

[b2-ehp-117-a140a] Choi H, Perera F, Pac A, Wang L, Flak E, Mroz E (2008). Estimating individual-level exposure to airborne polycyclic aromatic hydrocarbons throughout the gestational period based on personal, indoor and outdoor monitoring. Environ Health Perspect.

[b3-ehp-117-a140a] Fowler PA, Cassie S, Rhind SM, Brewer MJ, Collinson JM, Lea RG (2008). Maternal smoking during pregnancy specifically reduces human fetal desert hedgehog gene expression during testis development. J Clin Endocrinol Metab.

[b4-ehp-117-a140a] Neubert D, Tapken S (1988). Transfer of benzo(*a*)pyrene into mouse embryos and fetuses. Arch Toxicol.

[b5-ehp-117-a140a] Perera FP, Tang D, Tu YH, Cruz LA, Borjas M, Bernert T (2004). Biomarkers in maternal and newborn blood indicate heightened fetal susceptibility to procarcinogenic DNA damage. Environ Health Perspect.

[b6-ehp-117-a140a] Rice JM, Ward JM (1982). Age dependence of susceptibility to carcinogenesis in the nervous system. Ann N Y Acad Sci.

[b7-ehp-117-a140a] Soyka LF (1980). Hepatic drug metabolizing enzyme activity and tumorigenesis in mice following perinatal exposure to benzo(*a*)pyrene. Pediatr Pharmacol.

[b8-ehp-117-a140a] Withey JR, Shedden J, Law FC, Abedini S (1993). Distribution of benzo[*a*]pyrene in pregnant rats following inhalation exposure and a comparison with similar data obtained with pyrene. J Appl Toxicol.

